# Molecular Mechanisms of Acute Drug Toxicity in Polypharmacy: Analgesic–Psychotropic Interactions

**DOI:** 10.3390/ijms27167168

**Published:** 2026-08-11

**Authors:** Nikolina Rijavec, Boris Rijavec

**Affiliations:** 1 University Psychiatric Clinic Ljubljana, Chengdujska cesta 45, SI-1260 Ljubljana, Slovenia; 2 Department of Psychiatry, Faculty of Medicine, University of Ljubljana, Vrazov trg 2, SI-1000 Ljubljana, Slovenia; 3 Institute of Pharmacology and Experimental Toxicology, Faculty of Medicine, University of Ljubljana, Korytkova 2, SI-1000 Ljubljana, Slovenia; boris.rijavec@mf.uni-lj.si; 4 Department of Anaesthesiology and Surgical Intensive Care, University Medical Centre Ljubljana, Zaloška cesta 2, SI-1000 Ljubljana, Slovenia

**Keywords:** analgesics, psychotropic drugs, polypharmacy, acute toxicity, cytochrome P450, pharmacogenomics, serotonin toxicity, opioid-induced respiratory depression, hERG, mitochondrial dysfunction

## Abstract

Concurrent exposure to analgesic and psychotropic drugs is frequent in patients with pain, psychiatric comorbidity, frailty, or acute-care needs. The clinically important question is whether analgesics and psychotropic drugs act on the same metabolic, transporter, receptor, ion-channel, or cellular stress systems. This narrative mechanistic review discusses those points of contact. CYP-mediated inhibition or induction, phenoconversion, altered parent-to-metabolite ratios, and blood–brain barrier transporter effects can change both systemic and central exposure, particularly through CYP2D6, CYP3A4, CYP2C9, CYP2B6, and P-glycoprotein. Pharmacodynamic toxicity may involve serotonergic excess, opioid and GABAergic effects in respiratory-control networks, hERG/IKr-related loss of repolarization reserve, or dopamine D2 receptor blockade. Non-opioid analgesics and psychotropic background therapy add further pathways involving renal and gastrointestinal vulnerability, hematological toxicity, mitochondrial injury, and altered central nervous system function. At the cellular level, mitochondrial dysfunction, oxidative stress, calcium dysregulation, and endoplasmic reticulum stress are discussed primarily as mechanistic or preclinical contributors unless direct clinical evidence is available. Overall, analgesic–psychotropic co-exposure is presented as a clinically important example of pathway convergence, while pharmacogenomic and computational approaches are interpreted in relation to drug exposure, organ reserve, and patient-specific vulnerability.

## 1. Introduction

Polypharmacy is commonly defined as the concomitant use of several medicines, often five or more, but medication count alone does not explain why a regimen becomes toxicologically important. From a molecular perspective, polypharmacy is better viewed as a dynamic risk state in which several drug molecules converge on shared metabolic enzymes, transporters, receptors, ion channels, organelle stress pathways, and physiological reserve. This is especially relevant in older or medically complex patients, in whom adverse drug reactions, hospitalization, functional decline, and mortality depend on both drug exposure and patient vulnerability [1,2].

In this review, analgesic–psychotropic polypharmacy refers to concurrent exposure to at least one analgesic-related agent and at least one psychotropic agent, irrespective of the total number of medications. The term denotes a pharmacological co-exposure category and does not replace conventional numerical definitions of polypharmacy. In practice, at least one analgesic-related drug, such as an opioid, NSAID, APAP/paracetamol, tramadol, ketamine, metamizole, or a gabapentinoid, may be added to a patient already receiving an antidepressant, benzodiazepine, hypnotic, antipsychotic, mood stabilizer, or another centrally acting drug. Such prescribing is often necessary and clinically justified. The problem arises when several molecular and physiological layers of risk become active in the same patient at the same time. In such combinations, pharmacokinetic exposure, blood–brain barrier transport, receptor-level convergence, repolarization reserve, mitochondrial function, redox balance, and organ perfusion may all become clinically relevant.

More recent pharmacoepidemiological data make this risk clinically tangible rather than purely theoretical. In older nursing home residents, CYP2D6-metabolized opioids used together with CYP2D6-inhibiting antidepressants were associated with poorer pain-related outcomes and more opioid-related adverse outcomes than comparable combinations with CYP2D6-neutral antidepressants [3]. A similar pattern was observed for tramadol: concomitant use with CYP2D6-inhibiting antidepressants was associated with a higher seizure risk than tramadol use with CYP2D6-neutral antidepressants [4]. These data are important because the clinical signal can be linked to a relatively clear molecular explanation: CYP2D6 inhibition, altered metabolism, and the combined possibility of poorer therapeutic control and greater toxicity.

Several convergent toxicity phenotypes are especially relevant. At the receptor and circuit level, these include serotonergic overstimulation and opioid–GABAergic respiratory depression [5,6]. At the pharmacokinetic and ion-channel levels, altered parent-to-metabolite ratios may result from CYP- and UGT-dependent pathways, while blood–brain barrier transport and hERG/IKr inhibition may modify central exposure and cardiac repolarization vulnerability [7,8,9,10]. Non-opioid pathways include APAP-related mitochondrial injury, NSAID-related gastrointestinal and renal toxicity, and metamizole-associated hematological toxicity [11,12,13,14].

Much of the literature is opioid-centered, but analgesic–psychotropic toxicity is not limited to opioid-based practice. In European and pre-hospital settings, relevant analgesics also include piritramide, ketamine, APAP/paracetamol, NSAIDs, and metamizole. German physician-staffed air-rescue data illustrate this broader pattern: in pediatric trauma, ketamine, fentanyl, and piritramide were among the commonly administered analgesics, whereas metamizole and paracetamol were also used for non-trauma acute pain [15]. Another acute-care problem is incomplete recognition of chronic psychotropic exposure: routine analgesia may be added to pre-existing treatment with clozapine, lithium, valproate, carbamazepine, sedating antidepressants, antipsychotics, benzodiazepines, or antidementia drugs [16,17,18,19,20]. Other hidden psychotropic exposures, including trazodone-associated α1-adrenergic priapism, cholinesterase inhibitor–anticholinergic opposition, and memantine–ketamine NMDA receptor overlap, are clinically relevant boundary examples rather than core pathways developed in detail here [21,22,23,24,25].

Existing reviews have generally examined narrower components of this problem. Reviews of opioid drug–drug interactions have primarily focused on pharmacokinetic pathways, individual CYP or UGT mechanisms, or selected opioids [8,26], whereas other syntheses have concentrated on a single toxicity phenotype, such as opioid-associated serotonin toxicity [27], or on clinically manifested opioid interactions in a defined population, where direct outcome evidence remained largely case-based [28]. The present review adds a distinct multilevel framework that links altered systemic and central nervous system exposure to receptor and ion-channel convergence, cellular and organelle stress, organ vulnerability, and the final acute toxicity phenotype. Analgesic–psychotropic combinations are therefore presented not as uniquely toxic, but as a clinically important model of pathway convergence. The framework also distinguishes documented clinical outcomes from controlled human pharmacology, observational associations, preclinical findings, and mechanistic plausibility.

The review follows the progression from drug exposure and transport to receptor and ion-channel effects, cellular stress, organ vulnerability, and patient-specific modifiers.

### Literature Search and Selection Approach

This article was designed as a structured narrative mechanistic review rather than as a systematic or scoping review. Targeted searches were conducted in PubMed, Scopus, and Web of Science/Clarivate before manuscript submission in June 2026. The searches covered analgesic–psychotropic co-exposure, acute toxicity, pharmacokinetic and pharmacodynamic interactions, drug metabolism and transport, receptor and ion-channel mechanisms, cellular and organelle stress, pharmacogenomics, and computational interaction prediction. Publications from 2000 onward were prioritized, while earlier seminal studies were retained when necessary.

The searches retrieved 6429 database records before cross-database deduplication. PubMed and Scopus results were entered into a working evidence table containing 5857 row-level records. After deduplication and initial relevance screening, 3062 unique candidate records remained. This number represents the working evidence pool and not the number of studies formally included or cited in the review.

Sources were selected according to their relevance to acute toxicity, molecular mechanisms, drug interactions, or organ vulnerability. Evidence type was matched to the claim: clinical outcomes were preferentially supported by clinical studies or evidence syntheses, exposure-related claims by human pharmacokinetic studies, and molecular mechanisms by appropriate experimental studies. Case reports were used selectively for uncommon clinically manifested interactions. Candidate records were excluded when they were duplicates, outside the predefined acute-toxicity or mechanistic scope, focused primarily on chronic efficacy without relevant safety information, or lacked sufficient methodological or bibliographic detail for interpretation. During revision, additional targeted searches addressed gabapentinoids, cannabinoids, UGT-mediated opioid metabolism, serotonin-toxicity criteria, QT-risk classification, and previous related reviews. No formal risk-of-bias assessment, meta-analysis, or PRISMA-guided systematic-review process was undertaken.

**Figure 1:**
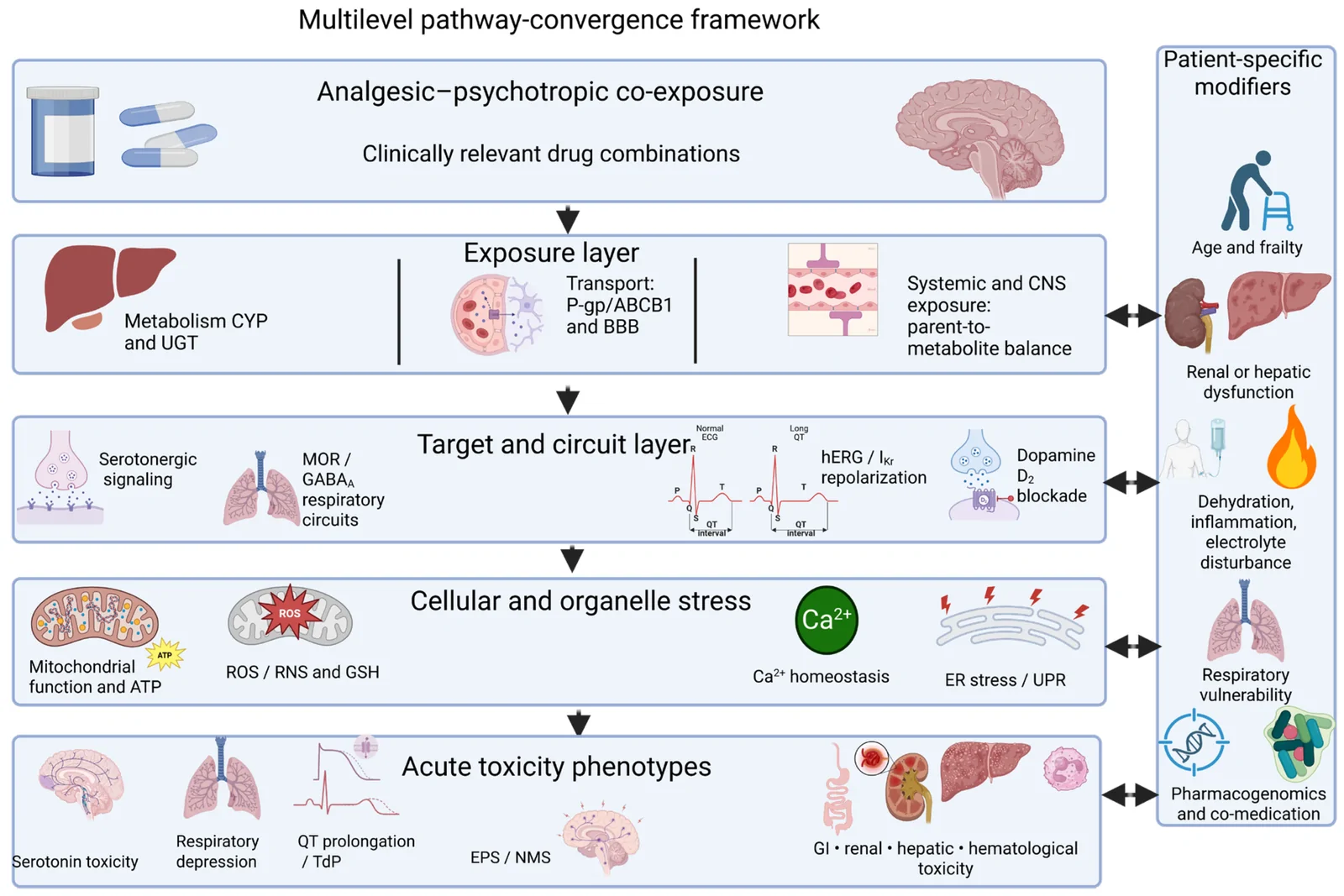
Multilevel pathway-convergence framework for acute toxicity during analgesic–psychotropic co-exposure. Pharmacokinetic processes may alter systemic and central nervous system exposure, followed by convergence at receptor, circuit, and ion-channel targets and by reduced cellular or organelle reserve. These interacting levels may contribute to acute neurological, respiratory, cardiac, gastrointestinal, renal, hepatic, or hematological toxicity. Patient-specific modifiers influence exposure, target-level effects, cellular resilience, and the resulting toxicity phenotype. The diagram is conceptual and does not imply a fixed linear sequence or an equal level of evidence for all pathways. Figure 1: Created in BioRender. Rijavec, B. (2026) https://BioRender.com/texpkeb (accessed on 7 August 2026).

Numerical estimates are presented as study-specific findings unless explicitly identified as pooled results, and the study design, population, and principal limitations are stated where relevant. The multilevel framework used in this review is summarized in Figure 1.

## 2. Molecular Pharmacokinetics

### 2.1. CYP450 and UGT Pathways, Phenoconversion, and Metabolic Crowding

Cytochrome P450 (CYP) enzymes are a major interface between analgesic and psychotropic drugs. For many drugs in both groups, CYP-mediated metabolism helps determine whether a compound is activated, inactivated, or cleared from circulation. When several such drugs are administered together, the same enzymatic system may be exposed simultaneously to substrates, inhibitors, active metabolites, and inducers. The interaction is therefore far from simple. It may involve competitive, non-competitive, or mixed inhibition, time-dependent inhibition, mechanism-based inactivation, and enzyme induction [29,30]. Among these enzymes, CYP2D6 and CYP3A4 are particularly important, although CYP2C9, CYP2C19, CYP1A2, CYP2B6, and UDP-glucuronosyltransferase pathways may also contribute, depending on the drug combination [7,8].

In this review, metabolic crowding is used as a descriptive, non-validated term for co-exposure to multiple substrates, inhibitors, inducers, or active metabolites that depend on overlapping metabolic pathways. It does not represent a separate molecular mechanism or a measurable pharmacokinetic parameter. Rather, it summarizes the combined effects of established processes such as competitive or time-dependent inhibition, enzyme induction, phenoconversion, and altered parent-to-metabolite ratios [29,30]. The term should not be interpreted as enzyme saturation unless saturation has been demonstrated experimentally.

The clinical meaning of CYP inhibition depends on the role of the inhibited metabolic pathway. If the pathway normally clears an active parent compound, inhibition may increase exposure and concentration-dependent toxicity; if the pathway is required for bioactivation, inhibition may instead reduce therapeutic response [29,30].

This distinction is particularly important for codeine and tramadol, because CYP2D6 contributes to the formation of their active opioid metabolites [7]. When CYP2D6 is inhibited by drugs such as fluoxetine, paroxetine, bupropion, or duloxetine, the patient may functionally resemble a CYP2D6 poor metabolizer, a process known as phenoconversion [31].

Phenoconversion may reduce opioid-mediated analgesia while increasing exposure to the parent compound, particularly tramadol [32]. Parent tramadol also inhibits serotonin and noradrenaline reuptake, providing a mechanistic link between altered metabolism, weaker opioid analgesia, serotonergic adverse effects, and seizure risk [27,32].

Clinically, concomitant use of CYP2D6-metabolized opioids with CYP2D6-inhibiting antidepressants has been associated with poorer pain-related and opioid-related outcomes, while tramadol combined with CYP2D6-inhibiting antidepressants has been associated with a higher seizure risk [3,4].

CYP3A4 adds another clinically relevant layer. Fentanyl and oxycodone are at least partly metabolized through CYP3A4, and the same enzyme is relevant for several benzodiazepines, hypnotics, antipsychotics, antidepressants, and other sedating drugs. CYP3A4 inhibition may increase exposure to compounds with sedative, respiratory depressant, or proarrhythmic effects, whereas induction may lower exposure and promote compensatory dose escalation or withdrawal-like effects. UGT-mediated glucuronidation provides an additional metabolic interface that should be considered alongside CYP pathways. Morphine is predominantly converted by UGT2B7 to morphine-3-glucuronide and the pharmacologically active morphine-6-glucuronide, while UGT-dependent pathways also contribute to the metabolism of oxycodone and oxymorphone, hydromorphone, codeine, dihydrocodeine, and buprenorphine [8,26]. Changes in glucuronidation may therefore alter parent-to-metabolite exposure and, depending on metabolite activity and elimination, modify analgesic or toxic effects [8]. However, many proposed UGT-mediated interactions, including inhibition by concomitant drugs, are supported mainly by in vitro or limited pharmacokinetic evidence and should not automatically be interpreted as clinically significant toxicity [8].

Pharmacotherapy with methadone or buprenorphine illustrates this interaction space because these opioids may be combined with benzodiazepines, antidepressants, antipsychotics, mood stabilizers, or enzyme-inducing drugs, leading to altered exposure, additive sedation, respiratory depression, withdrawal after changes in enzyme induction or inhibition, and QT-related risk [29,30,33,34,35].

Psychotropic background therapy can also create hidden pharmacokinetic baselines during acute care. Carbamazepine may reduce exposure to several antipsychotics through enzyme induction [19,36], whereas valproate has more complex, drug-specific, and smoking-dependent effects, including context-dependent changes in clozapine and norclozapine exposure [20,36]. Increased or reduced antipsychotic exposure may influence the response to analgesics, sedatives, ketamine, or antipsychotic rescue medication, especially when medication history is incomplete.

Pharmacologically defined cannabinoid medicines are used for distinct clinical indications and should not be treated as a uniform therapeutic class. Purified CBD is used for seizures associated with specific severe epilepsy syndromes, whereas dronabinol and nabilone are used for refractory chemotherapy-induced nausea and vomiting, and dronabinol for HIV/AIDS-related anorexia. Nabiximols, an approximately 1:1 THC–CBD oromucosal formulation, is approved in several countries for multiple-sclerosis-related spasticity. Cannabinoid-based products have also been evaluated for chronic pain, but the clinical benefit is generally small, formulation-dependent, and accompanied by neurological and psychiatric adverse effects [37].

Pharmacologically defined cannabidiol (CBD) is included here because it may modify exposure to psychotropic and analgesic-related drugs through CYP-mediated interactions; heterogeneous cannabis products are not treated as a uniform pharmacological category. CBD is metabolized mainly through CYP2C19 and CYP3A4 and may inhibit CYP2C19, CYP2D6, CYP2C9, and members of the CYP3 family, although many proposed interactions remain based on in vitro, preclinical, or indirect mechanistic evidence rather than demonstrated clinical outcomes [38,39]. In a small phase 2 pharmacokinetic study, highly purified CBD did not materially alter parent clobazam exposure but increased the maximum plasma concentration (Cmax) and the area under the plasma concentration–time curve over a dosing interval (AUCtau) of N-desmethylclobazam by 2.2-fold and 2.6-fold, respectively, consistent with CYP2C19 inhibition [40]. Concomitant valproate may increase the risk of CBD-associated hepatotoxicity, while cannabinoid products used with opioids, benzodiazepines, or sedating antidepressants may produce additive or synergistic CNS-depressant effects [37]. These findings should not be generalized to cannabis products containing variable proportions of CBD, THC, and other constituents, because composition, formulation, route of administration, dose, and metabolic effects differ substantially [39].

### 2.2. Transporter Dynamics: ABC Transporters, P-gp/ABCB1, and CNS Drug Exposure

Drug transporters are especially important for toxicity in the central nervous system. At the blood–brain barrier, P-glycoprotein (P-gp), encoded by the ATP-binding cassette subfamily B member 1 gene (*ABCB1*), is an ATP-dependent efflux transporter with a particularly important role. It limits the entry of many xenobiotics into the brain and thereby contributes to neural protection. In analgesic–psychotropic polypharmacy, plasma concentration alone may therefore not reflect clinically relevant exposure. The more relevant compartment may be the unbound drug concentration close to neuronal, glial, or brainstem receptor sites [9].

Several opioids interact with ABC transporters at the blood–brain barrier, but the evidence differs by drug. Loperamide is the clearest clinical example: under usual conditions it has minimal central opioid activity because P-gp restricts CNS penetration, whereas P-gp inhibition can allow central opioid effects, including respiratory depression [41,42]. For morphine, fentanyl, oxycodone, methadone, tramadol, and morphine glucuronides, the data are more heterogeneous and sometimes model- or species-dependent. Current evidence supports a more cautious conclusion: transporter activity can modify CNS opioid exposure, but P-gp does not uniformly determine toxicity for all opioids [9,43,44].

Transporter activity is dynamic. Pain, inflammation, chronic opioid exposure, withdrawal, nuclear receptor activation, and concomitant drugs may alter transporter expression or function. Some psychotropic drugs may also be P-gp substrates or modulators. Bidirectional interaction is therefore possible: a psychotropic drug may alter CNS access of an opioid, while opioid exposure, inflammation, or transporter induction may alter brain exposure to psychotropic drugs. These effects may not be obvious from plasma concentrations, especially when toxicity is driven by CNS receptor exposure [9,43,44].

Metabolism and transport should be interpreted together. Inhibition of a clinically relevant metabolic pathway may increase systemic exposure to the parent drug and alter parent-to-metabolite ratios, whereas inhibition of an efflux transporter at the blood–brain barrier may increase CNS exposure to susceptible substrates. Increased efflux-transporter expression or activity may reduce brain penetration and attenuate therapeutic effects, although the clinical importance of this mechanism is substrate- and context-dependent. In analgesic–psychotropic polypharmacy, acute toxicity may arise through linked events: reduced clearance, altered parent-to-metabolite ratios, changed brain penetration, and consequent excessive receptor-level or cellular toxicity. Pharmacokinetics is not merely a background topic; it is one of the molecular drivers of acute toxicity [7,9,29].

## 3. Pharmacodynamic Convergence: Receptor-, Circuit-, and Ion-Channel-Level Toxicity

In analgesic–psychotropic polypharmacy, toxicity may appear even when one drug does not substantially change the plasma concentration of another. The problem is often pharmacodynamic convergence. Different agents can act on the same physiological system, or reduce the reserve of that system. In this review, this issue is discussed mainly at four sites: serotonergic synapses, respiratory rhythm and arousal networks, cardiac repolarization, and dopamine D2 receptor pathways. These sites correspond to four acute toxicity patterns: serotonergic overstimulation, μ-opioid/GABAergic respiratory depression, hERG/IKr-mediated repolarization delay, and excessive D2 receptor blockade with extrapyramidal toxicity or neuroleptic malignant syndrome.

### 3.1. The Serotonergic Synapse: Molecular Triggers of Serotonin Toxicity

Serotonin toxicity is relevant to analgesic–psychotropic co-exposure because several analgesic-related drugs have serotonergic properties or become problematic when serotonergic psychotropics are already present. The syndrome reflects excessive serotonergic activity in the central and peripheral nervous systems. Clinically, it is usually recognized by a combination of mental-status change, autonomic activation, and neuromuscular hyperexcitability. Severe cases may progress to hyperthermia, rigidity, delirium, seizures, metabolic complications, and death [5,27,45].

Clinical recognition can be supported by the Hunter Serotonin Toxicity Criteria. In a patient exposed to a serotonergic agent, the criteria are fulfilled by spontaneous clonus; inducible clonus accompanied by agitation or diaphoresis; ocular clonus accompanied by agitation or diaphoresis; tremor with hyperreflexia; or hypertonia with a temperature above 38 °C and ocular or inducible clonus [46]. In the original study, these decision rules showed 84% sensitivity and 97% specificity relative to diagnosis by a clinical toxicologist [46]. Because the criteria were derived and evaluated in overdose cohorts and no independent diagnostic gold standard was available, their generalizability to therapeutic polypharmacy is less certain; they should therefore support rather than replace clinical assessment [46].

Several molecular routes can increase serotonergic tone. These include enhanced serotonin release, inhibition of serotonin reuptake, impaired serotonin metabolism, direct receptor stimulation, and pharmacokinetic inhibition that increases exposure to serotonergic drugs. In analgesic–psychotropic combinations, the most relevant analgesic-related agents are tramadol, meperidine, methadone, fentanyl, and dextromethorphan, especially when combined with SSRIs, SNRIs, tricyclic antidepressants, monoamine oxidase inhibitors, linezolid, methylene blue, or other serotonergic drugs [5,27,45].

Tramadol is a classic example of complex pharmacokinetics directly driving pharmacodynamics. CYP2D6 contributes to O-desmethyltramadol formation, and this metabolite is more important for opioid analgesia. However, parent tramadol also inhibits serotonin and noradrenaline reuptake. CYP2D6 inhibition by antidepressants such as fluoxetine, paroxetine, bupropion, or duloxetine may reduce opioid-mediated analgesia while increasing parent-drug exposure and monoaminergic burden. This pharmacokinetic shift helps explain why tramadol–CYP2D6-inhibiting antidepressant combinations have been associated with both reduced therapeutic control and acute CNS toxicity, including seizures or serotonin toxicity [4,7,27,31,32].

At the receptor level, no single serotonin receptor explains the full syndrome. Severe toxicity is often linked to 5-HT2A receptor activation, with possible contributions from 5-HT1A and peripheral serotonergic pathways. The 5-HT2A receptor is a Gq/11-coupled GPCR that activates phospholipase C-β, inositol 1,4,5-trisphosphate-mediated calcium release, protein kinase C, and downstream kinase signaling. This pathway provides a molecular bridge between excessive serotonergic stimulation and neuronal hyperexcitability. However, the clinical syndrome remains a network phenomenon involving serotonergic, monoaminergic, autonomic, spinal, cortical, and thermoregulatory effects [5,47].

Antidepressants contribute to analgesic–psychotropic toxicity through several distinct mechanisms and should not be treated as a pharmacologically uniform class. Strong or moderate CYP2D6 inhibition by agents such as fluoxetine, paroxetine, bupropion, or duloxetine may produce phenoconversion and alter parent-opioid-to-metabolite ratios; clinical studies have associated CYP2D6-inhibiting antidepressants with poorer outcomes during treatment with CYP2D6-metabolized opioids and, specifically for tramadol, with a higher seizure risk [3,4]. Pharmacodynamic risk also varies by class and drug: SSRIs, SNRIs, tricyclic antidepressants, and monoamine oxidase inhibitors can converge with serotonergic analgesic-related agents through different combinations of serotonin-reuptake inhibition, impaired monoamine metabolism, receptor-level effects, and altered drug exposure, although the strength of clinical evidence differs substantially between individual combinations [27]. Beyond serotonergic toxicity, SSRIs can reduce platelet serotonin availability and thereby amplify NSAID-related gastrointestinal bleeding risk [48,49], while selected antidepressants may contribute to loss of cardiac repolarization reserve, particularly at high exposure, in overdose, or when combined with other QT-prolonging drugs [10,50].

### 3.2. Ionic Interplay: μ-Opioid and Gamma-Aminobutyric Acid Type A (GABA-A) Receptor Crosstalk in Respiratory Depression

Respiratory depression is one of the most dangerous acute toxicities in analgesic–psychotropic co-exposure. Opioid-induced respiratory depression is primarily mediated by μ-opioid receptors (MORs). These are Gi/o-coupled GPCRs expressed in respiratory-control networks of the brainstem and pons, including the preBötzinger complex, ventral respiratory column, parabrachial/Kölliker-Fuse region, nucleus tractus solitarius, and related rhythm-generating or rhythm-modulating structures [51,52].

MOR activation inhibits adenylyl cyclase, reduces cAMP signaling, opens G-protein-coupled inwardly rectifying potassium channels, and inhibits presynaptic voltage-gated calcium channels. The result is neuronal hyperpolarization, reduced neurotransmitter release, impaired rhythm generation, and reduced ventilatory response to hypercapnia or hypoxia [51,53]. These effects become more dangerous when sedative psychotropics are added. Benzodiazepines, Z-drugs, barbiturates, and selected anesthetic agents enhance GABA-A receptor-mediated chloride conductance, increase inhibitory tone, and reduce arousal responses [6,54,55].

The opioid–benzodiazepine interaction should not be reduced to a vague “sedative burden.” Opioids suppress excitatory drive and respiratory rhythm, whereas GABAergic drugs increase inhibitory signaling and impair arousal. Together they can reduce respiratory frequency, tidal volume, upper-airway tone, and the ability to respond to rising CO2 or falling oxygen. Gabapentinoids represent a mechanistically distinct form of convergence. Although structurally related to GABA, gabapentin and pregabalin do not act as direct GABA-A receptor agonists; their principal analgesic effect is linked to binding to the α2δ-1 auxiliary subunit of voltage-gated calcium channels and reduced excitatory neurotransmitter release [56]. In a randomized, double-blind crossover study involving 12 healthy volunteers, pregabalin potentiated remifentanil-associated ventilatory depression: at the highest remifentanil target concentration, the increase in end-tidal carbon dioxide was 10.1 mmHg with remifentanil alone and 16.4 mmHg with pregabalin plus remifentanil. The combination also adversely affected cognitive performance [57]. This study-specific finding should not be extrapolated directly to routine clinical use. In a systematic review and meta-analysis, respiratory depression was not significantly increased in analyses restricted to randomized controlled trials, whereas non-randomized studies showed an association with respiratory depression (OR 1.71, 95% CI 1.31–2.24) and signals of increased mortality; however, the observational evidence was heterogeneous and had a substantial risk of bias [58]. Thus, controlled human pharmacology supports a potential gabapentinoid–opioid interaction, while the magnitude of real-world clinical risk is currently supported more strongly by observational than by randomized evidence.

Preclinical rat data support a pharmacodynamic buprenorphine–diazepam interaction. Diazepam enhanced sedation and respiratory depression without clearly changing buprenorphine brain kinetics or receptor binding under the tested conditions [6,54].

Patient context determines the threshold for toxicity. Sleep, obstructive sleep apnea, obesity, chronic lung disease, old age, frailty, renal impairment, alcohol, gabapentinoids, and other sedatives may reduce respiratory reserve or impair arousal [51,55]. In this setting, MOR activation increases K^+^ conductance and reduces Ca^2+^-dependent neurotransmitter release, while GABA-A modulation increases Cl^−^-mediated inhibition. The combined effect is reduced excitability in circuits that must remain responsive during sleep, sedation, hypercapnia, hypoxia, and airway compromise [6,51,55].

### 3.3. Ion Channel Interference: hERG Blockade and QT Interval Prolongation

Cardiac repolarization is another control point where analgesics and psychotropics may converge. The human ether-à-go-go-related gene potassium channel (hERG), encoded by KCNH2, conducts the rapid delayed rectifier potassium current (IKr). This current is essential for phase 3 ventricular repolarization. hERG inhibition reduces outward potassium current and delays repolarization. As a result, the QT interval may be prolonged and early afterdepolarizations may occur. In susceptible myocardium, these changes can promote torsades de pointes [10,59].

Methadone is the most relevant opioid example in this context, because it may be encountered both as an analgesic and as opioid agonist or maintenance therapy for opioid dependence. It is a μ-opioid receptor agonist, but it can also block hERG and prolong QT. This effect is stereoselective. S-methadone blocks hERG more potently than R-methadone, while R-methadone is more closely related to opioid agonism. CYP2B6 slow metabolizer status may increase S-methadone exposure, and this has been associated with longer QTc and greater QT-prolongation risk during racemic methadone therapy [34,35].

Opioids should not be regarded as a uniform class with respect to arrhythmogenic risk. The clearest and most consistent association with QT prolongation and torsades de pointes has been described for methadone. For tramadol and oxycodone, the risk appears less pronounced, but it may become relevant at higher exposure. For oxycodone, a dose-dependent association with QTc prolongation has been reported, together with low-affinity hERG inhibition in vitro; however, the available clinical evidence is associative rather than causal, and torsades de pointes has not been clearly established as a routine oxycodone-related event. For tramadol, dose was not associated with QTc prolongation in one small opioid-comparison study, whereas other data suggest that plasma concentration and reduced elimination, for example in renal impairment, may be more relevant. Morphine and buprenorphine are generally considered less problematic with respect to QT prolongation when used at usual therapeutic doses [34,60,61].

The combination of opioids with psychotropic drugs may further increase this risk when the psychotropic agent prolongs the corrected QT interval (QTc), reduces repolarization reserve, or has been associated with torsades de pointes. This risk should not be inferred from QTc length alone, because the relation between QT prolongation and torsades de pointes is imperfect and depends on drug-specific, patient-specific, and metabolic factors [10,50,62].

The risk is also heterogeneous across psychotropic drugs. Among antipsychotics, thioridazine, intravenous haloperidol, and ziprasidone are often regarded as higher-risk examples. Other clinically relevant agents include fluphenazine, oral or intramuscular haloperidol, risperidone, paliperidone, iloperidone, amisulpride, sertindole, pimozide, droperidol, chlorpromazine, and levomepromazine, particularly at higher doses, higher exposure, or in the presence of additional risk factors [50,62,63].

Quetiapine and clozapine should not be presented as carrying the same level of QT-related risk as thioridazine or intravenous haloperidol [62]. Nevertheless, they may become relevant in clinically vulnerable situations. For quetiapine, reported cases of torsades de pointes have mostly occurred in the setting of overdose, coadministration of other QT-prolonging drugs, electrolyte abnormalities, cardiac disease, female sex, or CYP3A4-mediated metabolic inhibition, rather than uncomplicated therapeutic use without additional risk factors [62,64].

Among antidepressants, QT liability is most often discussed for citalopram and escitalopram, tricyclic antidepressants, and selected agents such as venlafaxine or trazodone. The risk becomes more relevant at high concentrations, in overdose, during metabolic inhibition, or when several drugs affecting repolarization are combined. Overall risk is further increased by bradycardia, structural heart disease, hypokalemia, hypomagnesemia, toxic serum concentrations, renal or hepatic impairment, CYP-mediated inhibition of metabolism, and co-prescription of more than one QT-prolonging drug [10,50,62,65].

Drug-specific risk may be interpreted using the CredibleMeds/QTdrugs classification, which distinguishes drugs with known, possible, or conditional risk of torsades de pointes. Conditional risk refers to situations in which torsades de pointes occurs predominantly under defined circumstances, such as excessive exposure, electrolyte disturbance, or co-administration of interacting drugs [66,67]. These categories indicate the nature and strength of the available evidence but do not provide a quantitative ranking of risk within each category; the current online list should therefore be consulted because classifications may change as evidence is updated [66,67].

The key concept is repolarization reserve. A single mild hERG blocker may be tolerated, but risk rises when several stressors converge. Examples include two or more QT-prolonging drugs, metabolic inhibition, hypokalemia, hypomagnesemia, bradycardia, congenital susceptibility, structural heart disease, renal or hepatic impairment, and high drug concentration [10,50,62]. In a patient receiving methadone together with a QT-prolonging antipsychotic or antidepressant, arrhythmogenic risk may reflect direct hERG blockade, increased methadone exposure, impaired psychotropic clearance, reduced repolarization reserve, or all of these simultaneously [10,35,50,62].

### 3.4. Dopamine D2 Receptor Blockade, Extrapyramidal Toxicity, and Neuroleptic Malignant Syndrome

Antipsychotics add another pharmacodynamic control point: dopamine D2 receptor blockade. In acute-care practice, D2 blockade may enter analgesic care indirectly through antiemetic or procedural-sedation regimens, not only through chronic antipsychotic treatment. Dopamine-antagonist antiemetics and antipsychotics can share part of the same toxicity space: extrapyramidal symptoms, sedation, QT vulnerability, anticholinergic rescue medication, and rarely neuroleptic malignant syndrome [68,69].

Excessive D2 blockade, or D2 blockade in a vulnerable clinical context, can produce acute dystonia, akathisia, parkinsonism, or rigidity through nigrostriatal dopaminergic inhibition. Recent dose–response evidence supports a dose-dependent extrapyramidal-symptom gradient across antipsychotics. Risk rises substantially when estimated D2 receptor occupancy exceeds approximately 75–85%, although partial agonists and individual receptor profiles modify this relationship [68]. Binding kinetics should also be considered; experimental work suggests that D2 receptor association kinetics and receptor rebinding may contribute to extrapyramidal liability, not only static receptor occupancy [70].

D2-related motor toxicity has two implications for analgesic–psychotropic polypharmacy. First, motor toxicity may be misread as agitation, pain, anxiety, or delirium, leading to further medication layering. Second, treatment of acute dystonia with centrally acting anticholinergic drugs may increase anticholinergic burden [71,72]. In older or medically vulnerable patients, anticholinergic burden has been associated with delirium, especially when measured with anticholinergic risk scales [72]. An antipsychotic adverse effect and its pharmacological correction may therefore both add to acute neurotoxicity [71,72].

Neuroleptic malignant syndrome (NMS) represents the most severe manifestation of D2-related toxicity. NMS is rare but potentially life-threatening and classically involves hyperthermia, rigidity, altered mental status, and autonomic instability. Its pathophysiology is incompletely defined. Leading models include central D2 receptor blockade, impaired thermoregulation, basal-ganglia motor dysfunction, and possible direct skeletal-muscle toxicity. Reported risk factors include high antipsychotic dose, recent initiation or dose increase, parenteral administration, polypharmacy, dehydration, physical restraint, high ambient temperature, comorbidity, and previous NMS [69].

Together, serotonergic toxicity, respiratory depression, QT prolongation, extrapyramidal toxicity, and NMS show the same toxicological logic. Acute harm may arise when several drugs converge on receptor systems, ion channels, or neural circuits with limited reserve. Analgesic–psychotropic polypharmacy therefore requires a receptor- and ion-channel-level framework, not merely a list of drug pairs.

## 4. Cellular, Organelle, and Non-Opioid Analgesic Toxicity

In patients receiving analgesics together with psychotropic drugs, acute toxicity depends on several factors. Basic upstream processes such as receptor binding, ion-channel blockade, or altered plasma concentrations are transmitted to cellular stress systems. At this level, the cell either adapts or fails. The main factors influencing this outcome are mitochondrial function, redox balance, calcium regulation, glutathione availability, and endoplasmic reticulum (ER) stress [73,74,75,76]. These processes are important because they affect ATP production, membrane integrity, protein folding, inflammatory signaling, and cell-death thresholds. In polypharmacy, even several mild insults may become toxic when they affect the same vulnerable tissue or occur in a patient with reduced physiological reserve [73,74,75,76].

### 4.1. Mitochondrial Membrane Potential: Electron Transport Chain Disruption and ATP Depletion

Drug-induced toxicity often affects mitochondria. Their role goes well beyond ATP production. Mitochondria influence reactive oxygen species (ROS), buffer calcium, and participate in the regulation of cell death. When a drug affects the respiratory chain, mitochondrial membrane potential (ΔΨm), fatty-acid oxidation, or permeability transition, ATP availability may decrease. The cell may then shift from an adaptive stress response toward apoptosis, necrosis, or a mixed pattern of cellular injury [73,74].

This is most clearly seen with acetaminophen (APAP). In toxic exposure, APAP increases the formation of the reactive metabolite N-acetyl-p-benzoquinone imine (NAPQI). This depletes glutathione (GSH) and promotes the formation of mitochondrial protein adducts. These mechanisms may trigger mitochondrial oxidative stress, c-Jun N-terminal kinase activation, permeability transition, ATP depletion, and hepatocyte death [11,77,78]. APAP hepatotoxicity is usually presented as a single-drug overdose mechanism. The same mechanism is also useful as a model of reduced mitochondrial reserve. Factors that alter CYP activity or glutathione availability, including malnutrition, fasting, and chronic alcohol exposure, may modify susceptibility to APAP-induced injury [11,77,78].

Psychotropic drugs may add another layer of mitochondrial stress. In experimental conditions, antipsychotics and antidepressants have been associated with changes in mitochondrial respiration, oxidative stress, calcium regulation, and mitochondrial quality-control pathways. The clinical meaning of these findings depends on the drug, tissue, dose, and duration of exposure [79,80]. The main toxicological question is therefore not whether every analgesic–psychotropic pair directly injures mitochondria. The more important question is whether combined exposure reduces organelle reserve enough for a second stressor to become clinically relevant.

### 4.2. Oxidative Stress: Reactive Oxygen Species (ROS) Generation and Glutathione Depletion

Oxidative stress occurs when ROS or reactive nitrogen species exceed antioxidant and repair capacity. In acute drug toxicity, this imbalance may reflect reactive metabolite formation, impaired mitochondrial electron transport, inflammatory ROS production, reduced GSH availability, or impaired NADPH-dependent regeneration of reduced glutathione and thioredoxin by glutathione reductase and thioredoxin reductase, respectively [73,74,77].

These pathways are mechanistically interdependent: mitochondrial dysfunction may increase reactive oxygen species generation and ATP depletion, whereas oxidative and endoplasmic reticulum stress may further impair calcium handling and adaptive cellular responses [74,76,77,81]. However, evidence for this organelle-level convergence in analgesic–psychotropic co-exposure remains predominantly mechanistic or preclinical rather than clinically quantified [79,80,81].

### 4.3. Endoplasmic Reticulum Stress: Unfolded Protein Response (UPR) Activation and Drug-Induced Apoptosis

The ER is responsible for protein folding, lipid synthesis, calcium storage, and stress signaling. When misfolded proteins accumulate or ER calcium homeostasis is disrupted, the unfolded protein response (UPR) is activated through three principal sensors: protein kinase R-like endoplasmic reticulum kinase (PERK), inositol-requiring enzyme 1 alpha (IRE1α), and activating transcription factor 6 (ATF6). Initially, the UPR is adaptive: it reduces global translation, increases folding capacity, and promotes degradation of misfolded proteins. If stress persists, the same pathways may contribute to inflammation, mitochondrial dysfunction, calcium dyshomeostasis, and cell death through mediators such as activating transcription factor 4 (ATF4), C/EBP homologous protein (CHOP), X-box-binding protein 1 (XBP1), c-Jun N-terminal kinase (JNK), and regulated IRE1-dependent decay (RIDD), an IRE1α-mediated process that degrades selected cellular RNAs [76,82,83].

### 4.4. Non-Opioid and Acute-Care Analgesic Toxicity in Psychotropic Co-Exposure

NSAID–SSRI co-exposure is an important non-opioid example of acute toxicity in analgesic–psychotropic polypharmacy. Each drug class contributes through a different mechanism. NSAID-related upper gastrointestinal bleeding is mainly driven by COX-1/COX-2 inhibition, reduced synthesis of cytoprotective prostaglandins, impaired mucus and bicarbonate secretion, reduced mucosal blood flow, and delayed epithelial repair [12]. SSRIs act through platelet serotonin: by inhibiting serotonin reuptake into platelets, they reduce intraplatelet serotonin availability and may weaken platelet aggregation and hemostatic responses [48]. When both mechanisms are present, mucosal protection and hemostatic reserve may be jointly impaired [48].

This interaction is clinically relevant rather than only theoretical. A systematic review and meta-analysis found that, among patients already receiving NSAIDs, concomitant SSRI use was associated with a higher risk of upper gastrointestinal bleeding compared with NSAID use alone [49]. Earlier literature also suggested that the SSRI–NSAID combination may produce more than an additive risk in some datasets [48]. For the present review, this example is important because it broadens the toxicity framework beyond opioid-centered mechanisms: one drug class weakens mucosal protection, while the other weakens platelet-mediated hemostasis.

Ketamine and metamizole are useful examples for showing why a broader analgesic framework is needed. Ketamine is an NMDA receptor antagonist with analgesic, dissociative, and psychotomimetic properties. In polypharmacy, relevant concerns include altered arousal, sympathetic activation, emergence phenomena, and interactions with sedatives or pre-existing psychotropic vulnerability [84]. Ketamine is also metabolized through hepatic CYP pathways. In one human liver microsome study, N-demethylation to norketamine at therapeutic concentrations was attributed mainly to CYP3A4, with smaller contributions from CYP2B6 and CYP2C9 [85].

Metamizole/dipyrone is most relevant here because its main severe toxicity is rare but potentially life-threatening agranulocytosis. The pathomechanism remains incompletely defined and may involve immunologic or toxic mechanisms. The reaction appears largely dose-independent, although risk may increase with longer exposure, and rapid onset may occur after re-exposure [14]. Clozapine is also associated with rare but clinically important neutropenia and agranulocytosis, with genome-wide studies supporting involvement of the HLA region in clozapine-induced agranulocytosis [86]. In acute care, the key issue is therefore not necessarily a proven direct molecular interaction between clozapine and metamizole. More important is the recognition of convergence on the same severe hematological phenotype when metamizole is prescribed to a patient already exposed to clozapine [14,86]. This risk may be missed if chronic psychiatric treatment is unknown when analgesia is introduced.

NSAID-related renal injury provides another non-opioid model of acute toxicity. In older adults and in the presence of dehydration, chronic kidney disease, heart failure, liver disease, diuretic therapy, or renin–angiotensin system inhibition, glomerular filtration may become partly dependent on prostaglandin-mediated afferent arteriolar vasodilation [13]. NSAID-induced COX inhibition can remove this compensatory mechanism, reduce renal perfusion, and precipitate acute kidney injury (AKI) [13]; sedating psychotropic co-exposure may contribute indirectly through reduced oral intake, dehydration, or delayed recognition of illness. Population-based evidence supports the clinical relevance of this mechanism, with current NSAID exposure associated with increased odds of AKI, particularly in older adults and patients with chronic kidney disease [87].

Lithium provides a clinically important psychotropic example of analgesic-related pharmacokinetic toxicity mediated by reduced renal elimination. Lithium is not meaningfully metabolized and is eliminated almost entirely by the kidney; it is freely filtered and largely reabsorbed in parallel with sodium handling, mainly in the proximal tubule [88,89]. NSAIDs can increase lithium exposure by inhibiting cyclooxygenase-dependent prostaglandin synthesis, reducing afferent arteriolar vasodilation, decreasing glomerular filtration, and thereby reducing lithium excretion [13,16,89]. This risk is amplified by dehydration, sodium depletion, chronic kidney disease, older age, diuretics, renin–angiotensin system inhibition, and acute intercurrent illness [88,90]. The interaction is not uniform across all NSAIDs; higher doses and longer exposure appear more problematic, while acetylsalicylic acid and sulindac have been described as weaker or inconsistent interactors [89]. Lithium accumulation may present with gastrointestinal symptoms, coarse tremor, weakness, confusion, dysarthria, nystagmus, ataxia, myoclonus, seizures, or renal dysfunction [88].

A further acute-care hazard concerns clozapine-induced gastrointestinal hypomotility. Clozapine-treated patients may have marked and clinically silent colonic hypomotility: in an objective radiopaque-marker study, median colonic transit time was 104.5 h in clozapine-treated patients compared with 23 h in patients receiving other antipsychotics, 80% of clozapine-treated patients had hypomotility, and self-reported constipation had poor sensitivity for detecting delayed transit [91]. This is relevant when analgesics or adjunctive drugs are administered before chronic psychiatric medication is known. Opioids can further reduce gastrointestinal motility, and anticholinergic agents used for extrapyramidal symptoms, hypersalivation, nausea, or other acute indications may add to the same motility burden. In a register-based study of patients with schizophrenia, clozapine, opioids, anticholinergics, and tricyclic antidepressants were each associated with increased ileus risk, while clozapine and anticholinergics were associated with fatal ileus [92]. Thus, in emergency or postoperative care, an opioid or anticholinergic drug may not create a new toxicity pathway de novo but may contribute to acute or subacute decompensation of pre-existing clozapine-related gastrointestinal hypomotility into ileus, bowel obstruction, aspiration, ischemia, perforation, sepsis, or death [17,18]. Table 1 summarizes selected analgesic–psychotropic combinations, their molecular toxicity interfaces, evidence basis, mechanism-informed monitoring, and risk-mitigation principles.

## 5. Pharmacogenomics: Patient-Specific Genetic Modifiers of Toxicity

In analgesic–psychotropic toxicity, pharmacogenomics adds a patient-specific layer of risk. Inherited variation may influence drug activation, clearance, transport, receptor sensitivity, and immune-mediated susceptibility. In polypharmacy, however, genotype should not be treated as a fixed predictor of toxicity. Its clinical relevance depends on the context of each individual case. Important modifiers include dose, route of administration, organ function, inflammation, age, comorbidity, and concomitant medication. The most relevant pharmacogenomic markers are therefore those that modify exposure or tissue response at the molecular sites discussed above. These include CYP enzymes, transporters, receptors, cardiac ion channels, and immune-risk pathways.

CYP2D6 is the most clinically intuitive example. It contributes to the bioactivation of codeine and tramadol and to the metabolism of several antidepressants and antipsychotics. Poor metabolizers or patients who undergo phenoconversion because of CYP2D6 inhibitors may have reduced formation of active opioid metabolites, reduced analgesia, and altered parent-drug exposure. For tramadol, this may be especially relevant because parent tramadol also contributes to serotonin and noradrenaline reuptake inhibition, linking reduced opioid analgesia with potentially increased monoaminergic or seizure-related toxicity [3,4,7,31,32].

CYP2C19 and CYP2D6 influence exposure to some antidepressants and antipsychotics. For psychotropic drugs, however, genotype–concentration relationships are not equally well established across entire drug classes. They are most useful for individual compounds where the association is sufficiently reproducible and clinically measurable. Meta-analytic data indicate clinically relevant exposure increases in poor or intermediate metabolizers for aripiprazole, haloperidol, risperidone, escitalopram, and sertraline. For many other antidepressants and antipsychotics, the evidence base is less precise, so interpretation should rely more on the specific drug and the clinical context [94,95].

Other pharmacogenomic signals should also be considered, although they are less directly actionable for clinical decision making in acute analgesic–psychotropic toxicity. CYP2B6 variability may influence S-methadone exposure and QTc vulnerability during racemic methadone therapy [35,96]. Variants in OPRM1, COMT, and transporter genes such as ABCB1 may contribute to interindividual differences in opioid response, central nervous system exposure, or adverse effects [7,97]. However, their predictive value is generally weaker and more dependent on clinical context than the predictive value of CYP2D6 for codeine or tramadol [7]. Genetic variability may also influence susceptibility to QT prolongation, particularly when it alters exposure to hERG-blocking drugs [35,96]. In many cases, however, acquired factors determine whether inherited susceptibility becomes clinically visible. These include hypokalemia, bradycardia, renal or hepatic impairment, drug concentration, and co-prescribed hERG blockers [10]. Immune-mediated toxicity represents a different pharmacogenomic logic. Human leukocyte antigen variation has been implicated in several severe drug hypersensitivity syndromes and has also been investigated in clozapine-induced agranulocytosis and drug-induced liver injury. These associations are mechanistically important because they link drug exposure to antigen presentation, immune activation, and tissue-specific injury; however, their clinical utility varies across ancestry groups, drugs, and phenotypes [86,98].

## 6. Future Directions: Machine Learning and Molecular Modeling

Analgesic–psychotropic polypharmacy creates a prediction problem that conventional drug-pair interaction lists do not handle well. In the same patient, several factors may coexist, interact, or contribute independently: CYP inhibition, transporter modulation, receptor-level convergence, reduced organelle reserve, renal impairment, inflammation, pharmacogenomic susceptibility, and incomplete medication history. Computational toxicology can help organize this complexity. Its value, however, depends on whether models remain linked to interpretable molecular mechanisms rather than being used only as black-box risk scores.

Physiologically based pharmacokinetic (PBPK) and population pharmacokinetic models are most useful when the available data are detailed enough to link drug-specific properties with patient-specific physiology and support clinical interpretation. Dose and half-life alone are too limited for this purpose. A useful simulation should account for the determinants relevant to the clinical question, which may include absorption, distribution, metabolic route, transport, protein binding, renal and hepatic function, and genotype [99,100]. When these factors are explicitly defined, such models can estimate how exposure changes after CYP inhibition or induction, a shift in the parent compound-to-metabolite ratio, renal impairment, aging, or transporter modulation [99,100]. In the combinations discussed in this review, the clearest use cases are drugs for which exposure is closely linked to therapeutic response or toxicity. These include CYP2D6-dependent opioids [32], methadone [33,34,35], clozapine and valproate [20,36], carbamazepine [19], and similar agents. In such cases, a change in exposure may affect both therapeutic control and acute toxicity, making it clinically and pharmacologically relevant [32,99,100].

Machine learning and deep learning approaches offer a complementary strategy. Models based on molecular fingerprints, SMILES strings, graph neural networks, transcriptomic or pharmacovigilance features, and knowledge graphs can support early DDI screening or identification of hidden interaction patterns [101,102,103,104,105], as well as broader toxicity prediction, including hERG liability and hepatotoxicity [106,107,108]. These approaches may be useful for triage, especially when the interaction space is too large for manual review. However, they may be limited by training-data bias, under-representation of frail and multimorbid patients, sparse data for higher-order polypharmacy, lack of dose and time information, and weak representation of acute physiological states such as dehydration, inflammation, hypoxia, or renal failure [103,104,105].

QSAR and molecular-descriptor approaches can identify structural features associated with lipophilicity, ionization, BBB penetration, hERG binding, mitochondrial liability, or metabolic vulnerability. They are useful for hypothesis generation, but molecular structure alone cannot determine clinical toxicity. Dose, route, formulation, protein binding, active metabolites, transporter activity, renal and hepatic function, inflammation, pharmacogenomics, tolerance, sleep state, electrolyte balance, and co-medication determine whether a molecular liability becomes an acute toxic phenotype [106,107,108,109].

The most useful future direction is probably not a single universal prediction model, but a model structure that integrates several relevant layers. These include molecular mechanisms, exposure modeling, patient-specific susceptibility, and clinical context. PBPK and population pharmacokinetic models are better suited for interpretable exposure prediction, whereas machine learning models may help with early signal detection and risk prioritization [99,104,105]. In analgesic–psychotropic toxicity, future models will need to move beyond warnings based only on individual drug pairs. They should link molecular mechanisms and molecular interactions with the clinical factors described above. In practice, this means representing higher-order polypharmacy and including active metabolites, transporter effects, pharmacogenomics, organ function, frailty, inflammation, and acute-care modifiers. Such models should not be evaluated only by statistical performance. They should also be judged by whether they improve mechanistic understanding, identify modifiable toxicity pathways, and remain consistent with clinical feedback.

## 7. Conclusions

Acute toxicity during analgesic–psychotropic co-exposure is determined less by medication count than by convergence at metabolic enzymes, transporters, receptors, ion channels, and vulnerable organs. Human evidence supports clinically relevant exposure changes resulting from selected CYP-mediated interactions, whereas many proposed UGT-mediated interactions remain supported mainly by pharmacokinetic, in vitro, or mechanistic evidence [8,26,28]. Serotonergic toxicity is supported by the mechanistic and clinical literature [27], opioid–gabapentinoid ventilatory depression by controlled human and observational evidence [57,58], and opioid-related QT prolongation by drug-specific electrophysiological and clinical data [34,35,60]. NSAID–SSRI gastrointestinal bleeding and NSAID-related impairment of lithium elimination represent clinically documented non-opioid interactions [49,88,90].

In contrast, mitochondrial dysfunction, oxidative stress, calcium dysregulation, and endoplasmic reticulum stress currently provide predominantly mechanistic or preclinical explanations for how reduced cellular reserve may amplify toxicity [74,77,79,80,81]. Interpretation should therefore distinguish documented clinical outcomes from controlled human pharmacology, observational associations, and preclinical or in vitro findings. Pharmacogenomic and computational approaches may support exposure and toxicity prediction but still require clinically informed interpretation [94,99,107]. Analgesic–psychotropic combinations are therefore presented as a clinically important example of pathway convergence rather than as a uniquely hazardous drug pairing.

## Figures and Tables

**Table 1:** Mechanism-linked clinical translation for selected analgesic–psychotropic combinations. The table presents selected examples rather than an exhaustive interaction list. Monitoring and mitigation principles should be adapted to drug exposure, comorbidity, organ function, and the clinical setting.

Drug Combination	Molecular Interface and Toxicity Signal	Evidence Basis	Mechanism-Informed Monitoring	Risk-Mitigation Principle
Tramadol plus CYP2D6-inhibiting and/or serotonergic antidepressants	CYP2D6 phenoconversion may increase parent-tramadol exposure while reducing formation of its active opioid metabolite; serotonergic convergence may additionally increase seizure or serotonin-toxicity risk.	Observational clinical associations supported by pharmacokinetic and mechanistic evidence [3,4,27].	Assess analgesic response, mental status, neuromuscular hyperactivity, autonomic findings, and seizures after initiation or dose changes [4,27].	Review the combined CYP2D6 and serotonergic burden and consider an alternative analgesic or antidepressant when clinically appropriate [26,27].
Opioid plus gabapentin or pregabalin	α2δ-mediated reduction of excitatory neurotransmission may converge with opioid effects in respiratory-control and arousal networks.	Controlled human experimental evidence demonstrates potentiation of ventilatory depression, whereas real-world respiratory and mortality signals arise mainly from heterogeneous non-randomized studies; RCT-only respiratory findings were not significant [57,58].	Monitor sedation, cognition, respiratory rate, and oxygenation; closer observation is appropriate in patients with reduced respiratory or physiological reserve [57,58].	Use the lowest effective doses, reassess the need for additional sedatives, and increase monitoring during initiation or dose escalation [58].
Opioid plus benzodiazepine or sedating antipsychotic	μ-Opioid receptor effects may converge with benzodiazepine-enhanced GABA-A receptor-mediated inhibition or with broader CNS-depressant effects of sedating antipsychotics, reducing arousal and ventilatory responsiveness.	Clinical and mechanistic evidence is substantial for opioid–benzodiazepine co-exposure, whereas direct acute-toxicity evidence for individual antipsychotic–opioid pairs remains limited and confounded [54,93].	Assess consciousness, respiratory pattern, oxygenation, blood pressure, and the presence of additional central depressants [54,93].	Avoid unnecessary sedative overlap and interpret forensic or observational associations cautiously when direct causal evidence is absent [93].
Methadone plus a QT-prolonging psychotropic	Combined hERG/IKr inhibition and reduced repolarization reserve may increase susceptibility to QT prolongation and torsades de pointes.	Mechanistic, clinical electrophysiological, and evidence-classification data support a concentration- and context-dependent risk [10,35,60,66].	Consider the ECG, electrolytes, heart rate, renal and hepatic function, drug concentrations, and the total QT-active medication burden [10,66].	Correct modifiable risk factors and reduce avoidable exposure to multiple QT-prolonging drugs, particularly when additional susceptibility factors are present [10,66].
NSAID plus SSRI	NSAID-related mucosal injury may converge with reduced platelet serotonin-dependent hemostasis.	Clinical evidence syntheses support an increased gastrointestinal bleeding risk during combined exposure [48,49].	Assess gastrointestinal symptoms, bleeding history, age, previous ulcer disease, anticoagulant or antiplatelet therapy, and NSAID dose and duration [49].	Limit unnecessary NSAID exposure and consider patient-specific gastroprotection or an alternative analgesic strategy when bleeding vulnerability is increased [48,49].
NSAID plus lithium	NSAID-induced reduction in prostaglandin-mediated renal perfusion may decrease glomerular filtration and lithium clearance, thereby increasing lithium exposure.	Clinical pharmacokinetic reviews and population-level evidence support a clinically important but drug- and patient-dependent interaction [88,89,90].	Monitor lithium concentration, renal function, hydration status, and neurological or gastrointestinal symptoms after NSAID initiation or dose change [88,90].	Avoid unrecognized or prolonged NSAID exposure, use the lowest necessary exposure, and arrange earlier lithium and renal-function reassessment when co-administration is unavoidable [88,89].

## Data Availability

No new data were created or analyzed in this study. Data sharing is not applicable to this article.

## Abbreviations

The following abbreviations are used in this manuscript:

5-HT	5-hydroxytryptamine
5-HT1A	5-hydroxytryptamine receptor 1A
5-HT2A	5-hydroxytryptamine receptor 2A
ABC	ATP-binding cassette
ABCB1	ATP-binding cassette subfamily B member 1
AKI	acute kidney injury
APAP	acetaminophen
ATF4	activating transcription factor 4
ATF6	activating transcription factor 6
ATP	adenosine triphosphate
AUCtau	area under the plasma concentration–time curve over a dosing interval
BBB	blood–brain barrier
cAMP	cyclic adenosine monophosphate
CBD	cannabidiol
CHOP	C/EBP homologous protein
CI	confidence interval
Cmax	maximum plasma concentration
CNS	central nervous system
COMT	catechol-O-methyltransferase
COX	cyclooxygenase
CYP	cytochrome P450
D2	dopamine D2 receptor
DDI	drug–drug interaction
ER	endoplasmic reticulum
EPS	extrapyramidal symptoms
GABA	gamma-aminobutyric acid
GABA-A	gamma-aminobutyric acid type A receptor
GFR	glomerular filtration rate
GI	gastrointestinal
GPCR	G protein-coupled receptor
Gq/11	Gq/11 family G protein
GSH	glutathione
hERG	human ether-à-go-go-related gene potassium channel
HLA	human leukocyte antigen
IKr	rapid delayed rectifier potassium current
IRE1α	inositol-requiring enzyme 1 alpha
JNK	c-Jun N-terminal kinase
KCNH2	potassium voltage-gated channel subfamily H member 2
MOR	μ-opioid receptor
NADPH	reduced nicotinamide adenine dinucleotide phosphate
NAPQI	N-acetyl-p-benzoquinone imine
NMDA	N-methyl-D-aspartate
NMS	neuroleptic malignant syndrome
NSAID	non-steroidal anti-inflammatory drug
OPRM1	opioid receptor mu 1
OR	odds ratio
PBPK	physiologically based pharmacokinetic
PERK	protein kinase R-like endoplasmic reticulum kinase
P-gp	P-glycoprotein
QSAR	quantitative structure–activity relationship
QTc	corrected QT interval
RCT	randomized controlled trial
RIDD	regulated IRE1-dependent decay
RNS	reactive nitrogen species
ROS	reactive oxygen species
SMILES	simplified molecular-input line-entry system
SNRI	serotonin–noradrenaline reuptake inhibitor
SSRI	selective serotonin reuptake inhibitor
TdP	torsades de pointes
THC	Δ9-tetrahydrocannabinol
UGT	UDP-glucuronosyltransferase
UGT2B7	UDP-glucuronosyltransferase 2B7
UPR	unfolded protein response
XBP1	X-box binding protein 1
ΔΨm	mitochondrial membrane potential
